# miR-140 inhibits porcine fetal fibroblasts proliferation by directly targeting type 1 insulin-like growth factor receptor and indirectly inhibiting type 1 insulin-like growth factor receptor expression via SRY-box 4

**DOI:** 10.5713/ajas.19.0438

**Published:** 2019-11-12

**Authors:** Hongwei Geng, Linlin Hao, Yunyun Cheng, Chunli Wang, Wenzhen Wei, Rui Yang, Haoyang Li, Ying Zhang, Songcai Liu

**Affiliations:** 1 College of Animal Science, Jilin University, Changchun, Jilin 130062, China; 2 Five-Star Animal Health Pharmaceutical Factory of Jilin Province, Changchun, Jilin 130062, China

**Keywords:** miR-140, Type 1 Insulin-like Growth Factor Receptor (*IGF1R*), SRY-box 4 (*SOX4*), Proliferation

## Abstract

**Objective:**

This study aimed to elucidate the effect of miR-140 on the proliferation of porcine fetal fibroblasts (PFFs) and identify the target genes of miR-140 in PFFs.

**Methods:**

In this study, bioinformatics software was used to predict and verify target genes of miR-140. Quantitative polymerase chain reaction and western blot were used to detect the relationship between miR-140 and its target genes in PFFs. Dual luciferase reporter gene assays were performed to assess the interactions among miR-140, type 1 insulin-like growth factor receptor (*IGF1R*), and SRY-box 4 (*SOX4*). The effect of miR-140 on the proliferation of PFFs was measured by CCK-8 when PFFs were transfected with a miR-140 mimic or inhibitor. The transcription factor SOX4 binding to promoter of *IGF1R* was detected by chromatin immunoprecipitation assay (ChIP).

**Results:**

miR-140 directly targeted *IGF1R* and inhibited proliferation of PFFs. Meanwhile, miR-140 targeted transcription factor SOX4 that binds to promoter of porcine *IGF1R* to indirectly inhibit the expression of IGF1R. In addition, miR-140 inhibitor promoted PFFs proliferation, which is abrogated by SOX4 or IGF1R knockdown.

**Conclusion:**

miR-140 inhibited PFFs proliferation by directly targeting *IGF1R* and indirectly inhibiting IGF1R expression via SOX4, which play an important role in the development of porcine fetal.

## INTRODUCTION

Intrauterine growth restriction is a major problem in swine production since the associated low birth weight leads to high rates of pre-weaning morbidity and mortality plus permanent retardation of growth and development. Complex biological events affect fetal growth and development [1]. In previous studies, the insulin-like growth factor (IGF) system was closely correlated with fetal growth and development [2] and acted as proliferation and differentiation factors in cultured fetal cells and preimplantation embryos [3,4]. The components of IGF system include *IGF1* and *IGF2*, type 1 and type 2 IGF receptors, a family of six secreted IGF-binding proteins (IGFBPs) and IGFBP proteases [5]. Direct evidence of the importance of IGF system in the regulation of fetal growth was provided by a series of studies using gene knockout. For example, embryos of type 1 insulin-like growth factor receptor (*IGF1R*) knockout mice displayed severe lung hypoplasia and markedly underdeveloped diaphragms, leading to lethal neonatal respiratory distress. IGF1R, which is crucial for IGF signal transduction, is in concert with both IGF1 and IGF2 [6]. Moreover, IGF1R is a tyrosine kinase cell surface receptor which is involved in the regulation of cell growth and metabolism. Ligand binding induces activation of *IGF1R*, which leads to activation of the phosphorylated serine/threonine kinase (p-AKT) signaling pathways to promote cell proliferation [7].

MicroRNAs (miRNAs), a class of small and single-stranded non-coding RNAs containing approximately 22 nucleotides in length, act as the regulators to direct destruction or translational repression of their target mRNAs via binding to their 3′-untranslated region (3′UTR) [8]. miRNAs regulate the expression of a wide variety of target genes. Therefore, miRNAs are involved in a wide range of biological processes including cell proliferation, apoptosis, differentiation and migration [9,10]. Recently, mounting evidence indicated that miRNAs correlated with fetal growth and development of pigs. For example, miR-148a overexpression improved the early development of porcine somatic cell nuclear transfer embryos [11]. And miR-29a mediated the impairment of intestinal epithelial integrity induced by intrauterine growth restriction in pig [12]. More miRNAs that affected fetal growth and development of pigs need to be found in future research.

Previous studies suggested that miR-140 targeted *IGF1R* to suppress tumor growth and metastasis of non-small cell lung cancer [13]. Furthermore, loss of miR-140 contributed to the development of age-related osteoarthritis-like changes [14], which may be related to the effects of IGF1R. However, to our knowledge, whether miR-140 affects fetal growth of pigs remains unknown. In this study, we investigated the roles of miR-140 in porcine fetal fibroblasts (PFFs) proliferation and evaluated whether *IGF1R* and *SOX4* were the target genes of miR-140. Then we detected whether transcription factor SOX4 regulated the transcriptional activity of *IGF1R* promoter to enhance the effects of miR-140 on the expression of *IGF1R* and PFFs proliferation. Taken together, the aim of this study was to demonstrate a novel role of miR-140 as a suppressor of proliferation in PFFs and to provide a potential insight into affecting fetal growth of pigs by miRNAs.

## MATERIALS AND METHODS

### Reagents and animals

Cell culture-related chemicals and media were purchased from Life Technologies (Gaithersburg, MD, USA) unless otherwise stated. All animal treatment complied with a protocol that was approved by the Institutional Animal Care and Use Committee of Jilin University (Number of permit: 201812061).

### Cell preparation and cell culture

PFFs were derived from whole conceptuses of Large White pigs at day 30 of pregnancy [15]. Briefly, the heads and internal organs were removed from the conceptuses and washed three times with Ca^2+^ and Mg^2+^ free phosphate-buffered saline supplemented with 1% penicillin and streptomycin. Then, the remnants were minced and digested with 0.25% trypsin-ethylenediaminetetraacetic acid. Dulbecco’s modified Eagle’s medium (DMEM) with 10% fetal bovine serum (FBS) was added to the medium to inactivate the enzyme. The suspended cells were centrifugated (1,000×g) for 5 min and seeded into plastic culture dishes (Corning, New York, USA). The cells were cultured in DMEM supplemented with 10% FBS, 1 mM sodium pyruvate, 1% nonessential amino acids, and 10 mg/mL penicillin–streptomycin in a humidified atmosphere of 5% CO_2_ at 39°C. Unattached cells were removed and attached cells were further cultured until confluency. PFFs were stored in liquid nitrogen with a freezing medium, which containing 80% DMEM, 10% dimethyl sulfoxide (Sigma, St. Louis, MO, USA), and 10% FBS.

### DNA and RNA isolation and quantitative real-time polymerase chain reaction

The genomic DNA was extracted from PFFs according to the manufacturer’s introductions of Multisource Genomic DNA Miniprep Kit (Axygen, Shanghai, China). Total RNA was extracted from PFFs using TRIzol reagent (Invitrogen, Carlsbad, CA, USA). Total RNA concentration for each sample was quantified by NanoDrop 2000 (Thermo Scientific, Waltham, MA, USA). The cDNAs from 1,000 ng of total RNA were obtained by PrimeScript RT reagent kit (TaKaRa, Dalian, China) according to the manufacturer’s introductions. Quantitative real-time polymerase chain reaction (qRT-PCR) was performed using TransStart Green qPCR SuperMix (Transgen Biotech, Beijing, China) according to the manufacturer’s introductions. The primers were synthesized by Genewiz (Genewiz, Jiangsu, China) (Table 1). Gene expression levels were normalized to the expression of β-actin using the comparative Ct (2^−ΔΔCt^) method.

### Plasmid construction

psiCHECK-2 reporter vectors were constructed as follows. The fragments (90 bp) of wild-type IGF1R 3′ UTR, mutant type IGF1R 3′ UTR, wild-type SRY-box 4 (SOX4) 3′ UTR and mutant type SOX4 3′ UTR were synthesized by Genewiz (China) and contained *NotI* and *XhoI* restriction sites. Wild-type IGF1R 3′ UTR and SOX4 3′ UTR contain putative miR-140 binding sites. Mutant type IGF1R 3′ UTR and SOX4 3′ UTR mutant miR-140 binding sites contained the mutation of AACCACT to ATCGAGT. The fragments were respectively ligated into the psiCHECK-2 vector (Promega, Guangzhou, China) at corresponding restriction sites to generate four kinds of psiCHECK-2 reporter vectors (WT-IGF1R, Mut-IGF1R, WT-SOX4, and Mut-SOX4).

pGL4.2 reporter vectors were constructed as follows. Wild-type *IGF1R* promoter fragments containing the binding site of SOX4 were amplified by PCR with primer WT, which contains a *XhoI* site (CTCGAG) and an *EcoRV* site (GATATC) as underlined in Table 1. Mutant-type *IGF1R* promoter fragments, whose binding site of SOX4 were mutated from CC AAAACAAGGGCGAA to CGATATCTACGCCCAT, were synthesized by Genewiz (Genewiz, China) and contained a *XhoI* site (CTCGAG) and an *EcoRV* site (GATATC) at each end, respectively. The products were obtained and cloned into corresponding sites of pGL4.20-basic vector (Promega, Madison, WI, USA) to generate pGL4.2 reporter vectors (WT-Promoter and Mut-Promoter).

SOX4 expression vectors (pcDNA3.1-SOX4) were constructed as follows. The coding sequence of *SOX4* was amplified by PCR with cDNA synthesized from PFF with primer_SOX4-cds_ containing a *XhoI* site (CTCGAG) and an *EcoRI* site (GAATTC) as underlined in Table 1. The PCR product was cleaved and cloned into corresponding sites of the pcDNA3.1 vector.

All clones were subjected to sequencing analysis (Genewiz, China) after construction.

### Cell transfection and Dual-luciferase reporter assays

PFFs (5×10^3^) were seeded in each well of 96-well plates with growth medium without antibiotics at 1 d before transfection to reach a confluency of 80% at transfection. 100 nM of miR-140 mimic, miR-control, miR-140 inhibitor, inhibitor control, siRNA for *SOX4* (si-SOX4), siRNA for *IGF1R* (si-IGF1R) or siRNA control (si-control) (GenePharma, Shanghai, China) (Table 2) were transfected into PFFs (100 μL medium), respectively, using Lipofectamine 2000 reagent (Invitrogen, USA) according to the manufacturer’s protocol.

To investigate the target genes of miR-140, the PFFs (100 μL medium) were cotransfected with the dual-luciferase reporter vectors (180 ng of WT-IGF1R, Mut-IGF1R, WT-SOX4, or Mut-SOX4, respectively, and 20 ng of Renilla luciferase vector) and 100 nM of miR-140 mimic or miR-140 inhibitor. After 48 h transfection, cells were collected for luciferase activity assays with the Dual-Luciferase Reporter Assay System (Promega, USA) according to the manufacturer’s protocol.

To detect the effects of SOX4 on the transcriptional activity of IGF1R promoter, 200 ng of pcDNA3.1-SOX4 or pcDNA3.1-control were cotransfected with WT-Promoter or Mut-Promoter into PFFs (100 μL medium). 48 h after transfection, the luciferase activity was measured.

### Cell proliferation assays

The 96-well plates were incubated at 39°C and proliferation of PFFs was assayed at 24, 48, and 72 h after transfection using a CCK-8 kit (Dojindo Laboratories, Kumamoto, Japan) according to the manufacturer’s instructions. Briefly, CCK-8 solution was added in each well and incubated for 1 h at 37°C. The optical density was measured at 450 nm using Victor3TM multilabel plate counter (PerkinElmer, Waltham, MA, USA).

### Western blotting assays

PFFs were thoroughly minced in lysis buffer contained 0.1 mM phenylmethylsulfonyl fluoride (PMSF) (Beyotime, Shanghai, China). Lysates (25 μg) were separated on sodium dodecyl sulfate (SDS)–polyacrylamide gel electrophoresis and then electrotransferred to nitrocellulose membranes (Whatman, Maidstone, UK). Membranes were blocked for 2 h at room temperature with 5% nonfat dried milk solution and then immunoblotted overnight at 4°C with primary antibodies against SOX4, IGF1R, and β-actin (1:2,000, Bioworld Technology CO., Ltd., Nanjing, China). After washing, the membranes were probed with HRP-conjugated secondary antibodies (1:2,000, Bioworld Technology CO., Ltd., USA). The enhanced chemiluminescence plus Western blotting detection system (Amersham Biosciences, Little Chalfont, Buckinghamshire, UK) was used for protein detection.

### Chromatin immunoprecipitation

Chromatin immunoprecipitation (ChIP) assay was performed according to the protocol of ChIP Assay Kit (Beyotime, Nanjing, China). In brief, cells were cross-linked with 37% formaldehyde for 10 min at room temperature. The reaction was stopped by 125 mM glycine for 5 min at room temperature. The cells were scraped and lysed in the SDS lysis buffer containing 1 mM PMSF. The lysate was sonicated and centrifugated. Then, the supernatant was immunoprecipitated overnight at 4°C with antibody anti-SOX4 (1:100, Cell Signaling Technology, Danvers, MA, USA). An equal mass of Normal Rabbit immunoglobulin G (1:1,000, Cell Signaling Technology, USA) was used as negative control. Following immunoprecipitation, the supernatant was transferred to protein A/G-agarose beads and rotated at 4°C for 45 min. The DNA was collected from beads and used as the template for real-time PCR using Primer_ChIP_ (Table 1). The relative quantity of DNA was determined by comparing the sample fluorescence to the fluorescence values measured from the total chromatin input dilution series.

### Bioinformatics method and statistical analysis

The miR-140 targets were predicted by TargetScan (http://www.targetscan.org/vert_72/). Transcription factors binding sites were predicted with Jaspar database (http://jaspar.genereg.net/). Data were expressed as the mean±standard deviation from at least three independent experiments. Differences among groups were tested by one-way analysis of variance and p<0.05 was considered statistically significant. Comparisons between two groups were evaluated using least significant difference t-test.

## RESULTS

### miR-140 directly targeted IGF1R to inhibit PFFs proliferation

**Figure 1:**
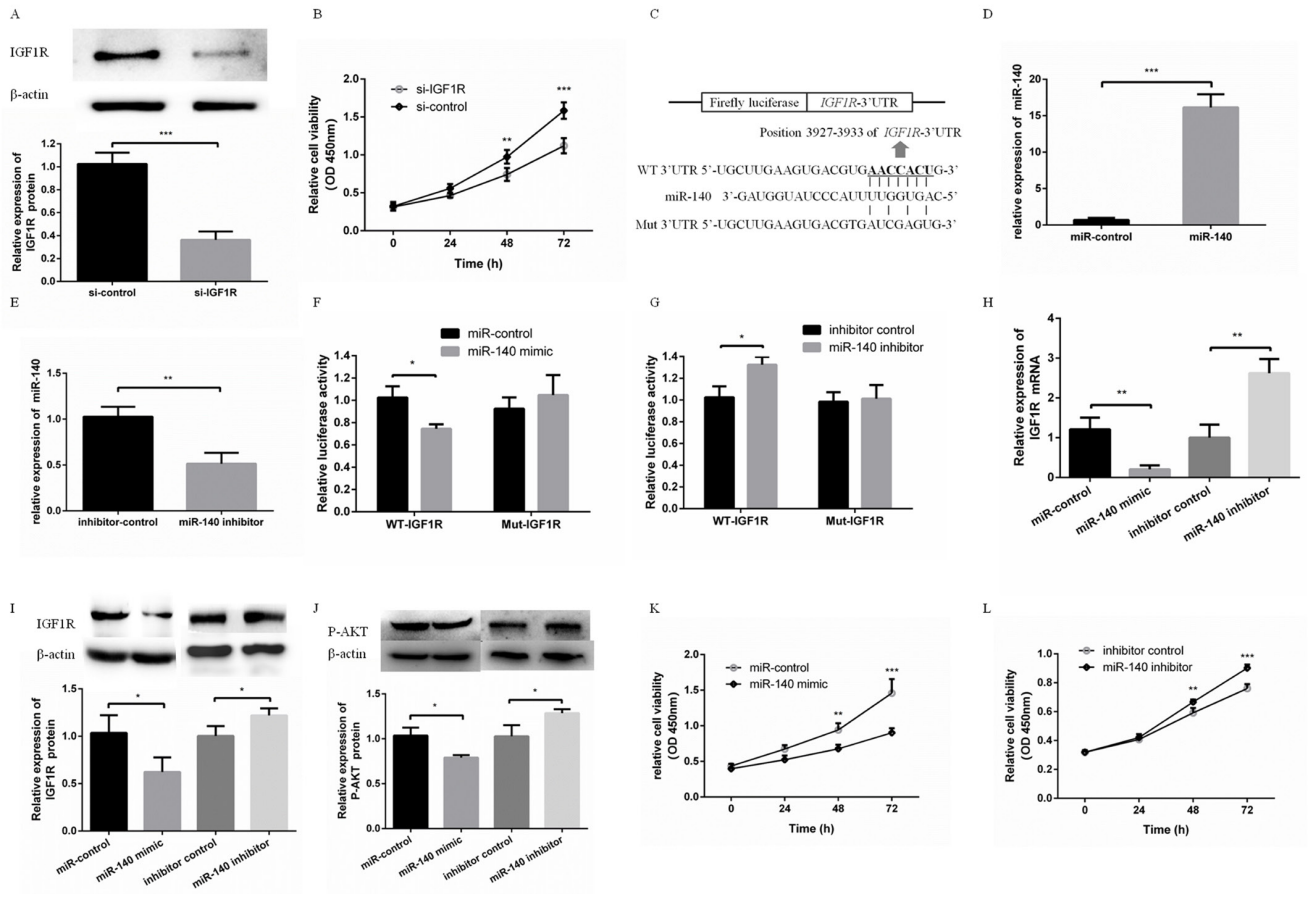
miR-140 directly targeted *IGF1R* to inhibit PFFs proliferation. (A) The protein expression level of IGF1R was measured at 48 h after transfecting with si-IGF1R. (B) PFFs proliferation was measured at 24, 48, and 72 h after transfecting with si-IGF1R. (C) Putative binding sequences of miR-140 and mutational binding sequences of miR-140 in the IGF1R 3′-untranslated region were listed. (D, E) The mRNA expression level of miR-140 was measured when PFFs were transfected with miR-140 mimic or miR-140 inhibitor. (F) miR-140 mimic was cotransfected with WT-IGF1R or Mut-IGF1R. Luciferase activity was measured at 48 h post-transfection. (G) miR-140 inhibitor was cotransfected with WT-IGF1R or Mut-IGF1R. Luciferase activity was measured at 48 h post-transfection (H, I). The mRNA and protein expression levels of IGF1R in PFFs were measured by qRT-PCR and Western blotting when PFFs were transfected with miR-140 mimic or miR-140 inhibitor, respectively. (J) Phosphorylation level of AKT was measured by Western blotting. (K) PFFs proliferation was measured at 24, 48, and 72 h after overexpression of miR-140. (L) PFFs proliferation was measured at 24, 48, and 72 h after transfecting with miR-140 mimic or miR-140 inhibitor. *IGF1R*, type 1 insulin-like growth factor receptor; PFF, porcine fetal fibroblast; qRT-PCR, quantitative real-time polymerase chain reaction. * p<0.05, ** p<0.01, *** p<0.001.

To detect the effects of IGF1R on PFFs proliferation, si-IGF1R was transfected into PFFs to inhibit the expression of IGF1R. Western blotting showed that si-IGF1R effectively inhibited the protein expression level of IGF1R (Figure 1A). The CCK8 assay showed PFFs proliferation was effectively inhibited via knockdown of IGF1R (Figure 1B). In addition, miR-140 was predicted to bind with IGF1R 3′UTR (Figure 1C). miR-140 mimic or miR-140 inhibitor were used to overexpress or suppress the expression of miR-140 in PFFs, respectively (Figure 1D, E). To validate this prediction, WT-IGF1R or Mut-IGF1R was cotransfected with miR-140 mimic or miR-140 inhibitor into PFFs. The luciferase activity of WT-IGF1R was significantly suppressed in PFFs transfected with miR-140 mimic, while increased in PFFs transfected with miR-140 inhibitor (Figure 1F). However, the luciferase activity of Mut-IGF1R was not significantly changed in PFFs transfected with miR-140 mimic or miR-140 inhibitor (Figure 1G). IGF1R mRNA level and protein level were suppressed (or increased) in PFFs transfected with miR-140 mimic (or miR-140 inhibitor) (Figure 1H, I). These results suggested that *IGF1R* was a direct target gene of miR-140. Furthermore, *IGF1R* is a strong activator of the phosphatidylinositol-4,5-bisphosphate 3-kinase (PI3K)-Akt pathway. The evolutionarily ancient IGF1R-PI3K-AKT pathway plays a key role in regulating cell proliferation [16–18]. In our study, overexpression of miR-140 increased AKT phosphorylation (P-AKT) level and inhibition of miR-140 decreased P-AKT level (Figure 1J). Overexpression of miR-140 suppressed proliferation of PFFs (Figure 1K). In contrast, knockdown of miR-140 promoted proliferation of PFFs (Figure 1L). This suggested that PI3K-AKT pathway was associated with miR-140 inhibiting PFFs proliferation. Taken together, these results demonstrated that miR-140 was able to inhibit PFFs proliferation by targeting directly to IGF1R.

### miR-140 targeted SOX4 to inhibit IGF1R expression indirectly

**Figure 2:**
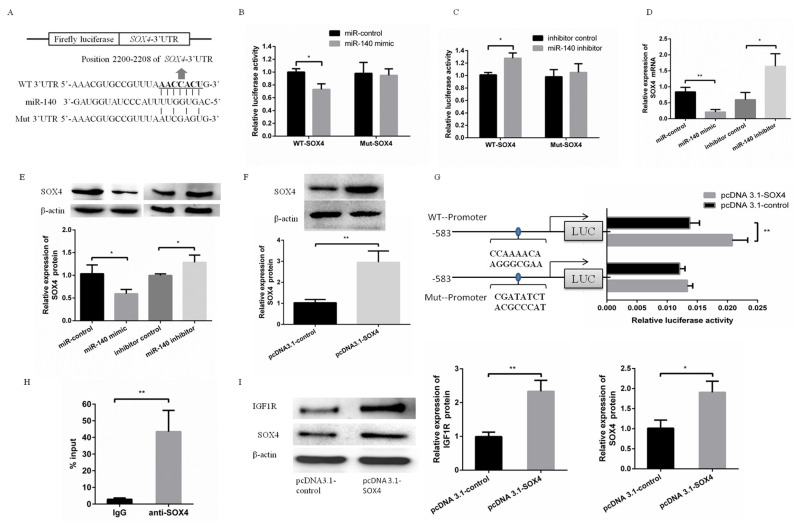
miR-140 directly targeted *SOX4* to inhibit IGF1R expression indirectly. (A) Putative binding sequences of miR-140 and mutational binding sequences of miR-140 in the SOX4 3′-untranslated region were listed. (B) miR-140 mimic was cotransfected with WT-SOX4 or Mut-SOX4. Luciferase activity was measured at 48 h post-transfection. (C) miR-140 inhibitor was cotransfected with WT-SOX4 or Mut-SOX4. Luciferase activity was measured at 48 h post-transfection. (D,E) The mRNA and protein expression levels of SOX4 in PFFs were measured by qRT-PCR and Western blotting when PFFs were transfected with miR-140 mimic or miR-140 inhibitor, respectively. (F) The protein expression level of SOX4 in PFFs transfected with pcDNA3.1-SOX4 was measured by Western blotting at 48 h post-transfection. (G) Putative binding site of SOX4 in the promoter of porcine IGF1R was listed. The WT-Promoter or Mut-Promoter was cotransfected with pcDNA3.1-SOX4 or pcDNA3.1-control into PFFs. And the luciferase activity was measured at 48 h post-transfection. (H) Putative binding site of SOX4 in the promoter of porcine IGF1R was detected by ChIP analysis. (I) The protein expression levels of SOX4 and IGF1R were detected by Western blotting when miR-140 was cotransfected with pcDNA3.1-SOX4 or pcDNA3.1-control into PFFs. *SOX4*, SRY-box 4; IGF1R, type 1 insulin-like growth factor receptor; PFF, porcine fetal fibroblast; qRT-PCR, quantitative real-time polymerase chain reaction. * p<0.05, ** p<0.01.

Meanwhile, *SOX4* was predicted as a target gene of miR-140 (Figure 2A). This prediction was validated by the same way with validating *IGF1R* as a target gene of miR-140. To validate this prediction, WT-SOX4 or Mut-SOX4 was cotransfected with miR-140 mimic or miR-140 inhibitor into PFFs. The luciferase activity of WT-SOX4 was significantly suppressed in PFFs transfected with miR-140 mimic, while increased in PFFs transfected with miR-140 inhibitor (Figure 2B). However, the luciferase activity of Mut-SOX4 was not significantly changed (Figure 2C). Furthermore, SOX4 mRNA level and protein level were suppressed (or increased) in PFFs transfected with miR-140 mimic (or miR-140 inhibitor), respectively (Figure 2D, E).

Using bioinformatic prediction, *IGF1R* was predicted to be a potential target of transcription factor SOX4. To detect the effects of SOX4 on transcriptional activity of *IGF1R* promoter, pcDNA3.1-SOX4 was cotransfected with pGL4.2-WT or pGL4.2-Mut. The luciferase activity showed overexpression of SOX4 increased transcriptional activity of *IGF1R* promoter. However, the effect of SOX4 on *IGF1R* promoter was abrogated when binding site of SOX4 in *IGF1R* promoter was mutated (Figure 2F). Meanwhile, ChIP assay confirmed the SOX4 bind with IGF1R promoter (Figure 2G). These data suggested that transcription factor SOX4 bind to *IGF1R* promoter to improve transcriptional activity of *IGF1R* promoter. To investigate the effect of transcription factor SOX4 on miR-140 inhibiting IGF1R expression, miR-140 mimic was cotransfected into PFFs with pcDNA3.1-SOX4 or pcDNA3.1-control, respectively. Our results showed that SOX4 abrogated the effect of miR-140 on IGF1R expression and played a key role in miR-140 inhibiting PFFs proliferation.

### miR-140 inhibitor promoted PFFs proliferation, which is abrogated by SOX4 or IGF1R knockdown

**Figure 3:**
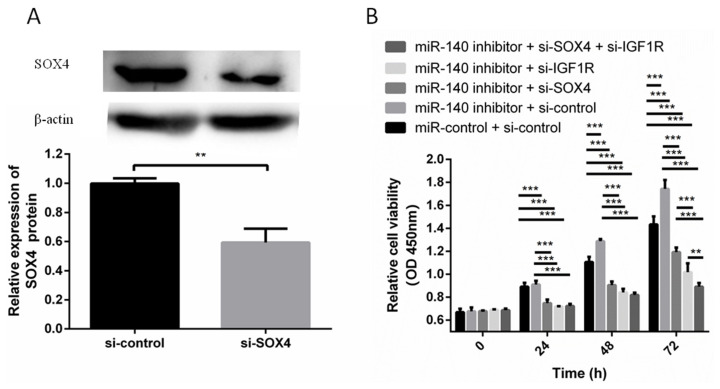
miR-140 inhibitor promoted PFFs proliferation, which was abrogated by SOX4 or IGF1R knockdown. (A) The protein expression level of SOX4 was detected by Western blotting when si-IGF1R was transfected into PFFs. (B) PFFs proliferation was measured at 24, 48, and 72 h after transfection. PFF, porcine fetal fibroblast; SOX4, SRY-box 4; IGF1R, type 1 insulin-like growth factor receptor. ** p<0.01, *** p<0.001.

Next we examined whether SOX4 and IGF1R were involved in the effect of miR-140 on PFFs proliferation. The expression level of SOX4 was suppressed by si-SOX4 in PFFs. Western blotting showed that si-SOX4 effectively inhibited the protein expression level of SOX4 (Figure 3A). miR-140 inhibitor was cotransfected with si-SOX4, si-IGF1R, or si-control into PFFs. CCK8 assays results showed that miR-140 inhibitor induced PFFs proliferation, which was abrogated by si-SOX4 and si-IGF1R (Figure 3B). So SOX4 and IGF1R reversed the effects of miR-140 on PFFs proliferation, which suggested that miR-140, promoted PFFs proliferation by targeting SOX4 and IGF1R.

## DISCUSSION

Among livestock animals, pigs exhibited the highest rates of intrauterine growth restriction [19,20]. A variety of factors, including changes of environmental temperature, feed hygiene and safety, suboptimal nutrition and disease, affected the fetal growth of pigs [21]. These negatively impact on the pigs and can last for their entire life and be carried over to the next generation or beyond [22]. Thus, it is of great significance to understand the fetal growth and development. Previous studies showed that IGF system played a key role in fetal growth and were regulated by some different factors. miRNAs, which are implicated in cell proliferation, differentiation and apoptosis and function as either suppressors or activators, play important roles in growth and development of pigs [23–25]. Previous studies focused on role of miR-140 in arthritis and various cancers. To date, however, the role of miR-140 in growth of pigs and the molecular mechanisms by which miR-140 exerts its functions remain unclear. In this study, we showed that miR-140 effectively inhibited PFFs proliferation via directly targeting *IGF1R*. Clinical data and *in vitro* studies demonstrated that the expression of the *IGF1R* gene had a profound effect on prenatal growth in humans and animals. Meanwhile, miR-140 target *SOX4* to indirectly inhibit IGF1R expression. This enhanced the effects of miR-140 inhibiting IGF1R expression. These results suggested that miR-140 was a regulator of fetal growth and development in pigs.

To elucidate the underlying mechanisms involved in the miR-140-induced inhibition on proliferation of PFFs, *IGF1R* and *SOX4* were identified as critical targets of miR-140. This conclusion was supported by the following evidences: i) Complementary sequence of miR-140 is identified in the 3′ UTR of *IGF1R* and *SOX4*; ii) Overexpression of miR-140 significantly reduced IGF1R and SOX4 level in PFFs; iii) Overexpression of miR-140 reduced the activity of a luciferase reporter containing the wild type 3′ UTR of *IGF1R* and *SOX4*, but failed to change the activity of a luciferase reporter containing the mutant type 3′ UTR of *IGF1R* and *SOX4*; iv) the inhibitory effects of miR-140 on proliferation of PFFs were reversed by knockdown IGF1R and SOX4. Taken together, these data strongly suggested that *IGF1R* and *SOX4* are the target genes of miR-140.

The transcription factor SOX4 belongs to a large family of proteins that regulate numerous aspects of embryonic development, such as retinal differentiation, central nervous system, heart development, and lymphocyte development, via its transcriptional activation [26,27]. SOX4 is structurally characterized by a highly conserved HMG-box domain that directly binds to the minor groove of DNA helix to generate a conformation that facilitates various DNA-dependent activities [28]. SOX4 binds to the promoters of different genes and affects the expression of these genes, thereby affecting the downstream pathways of these genes. For example, in acute lymphoblastic leukemia (ALL), mouse genetic studies have also demonstrated that SOX4 binds to and transcriptionally activated promoters of multiple components within the PI3K/AKT and MAPK signaling pathways, thereby affecting of PI3K/AKT and MAPK signaling in ALL cells [29]. In our study, SOX4, as a putative target of miR-140, was inhibited by miR-140 overexpression. Furthermore, we found that SOX4 bind to the promoter of porcine *IGF1R* via ChIP assay and dual-luciferase reporter gene assay. This suggested that SOX4 binds to the promoter of porcine *IGF1R* to enhance *IGF1R* expression and regulate PFFs proliferation. Overexpression of SOX4 abrogated the inhibition of miR-140 to IGF1R, indicating that SOX4 plays an important role in the inhibition of IGF1R expression by miR-140.

In summary, miR-140 inhibited PFFs proliferation by directly targeting *IGF1R*. In addition, miR-140 target *SOX4* to indirectly inhibit *IGF1R* expression. SOX4 enhanced the effects of miR-140 inhibiting IGF1R expression to inhibit PFFs proliferation. Our study suggested that miR-140 was a regulator of fetal growth and development in pigs.

## Figures and Tables

**Table 1:** Primer sequences

Name	Sequence (5′-3′)	Product size (bp)	Tm (°C)
*IGF1R*-QPCR-F	AGGAGAAGCCGCTGTGTGA	179	58
*IGF1R* -QPCR-R	TGTGTCGTTGTCGGGTGC		
*SOX4*-QPCR-F	GAACGCCTTCATGGTGTGGT	191	60
*SOX4*-QPCR-R	TGTAGTCGGGGTAGTCAGCCA		
β-actin -QPCR-PF	CCCGGAAACGCTCTTCCA	86	60
β-actin -QPCR-PR	CGCACTTCATGATGCTGTTGA		
WT F	CTCGAG CCGCTTTGTGTGTGTCCTG	582	58
WT R	GATATC CGAAATTCCCCTTTCTCAAAA		
*SOX4*-cds -F	GAATTCCCAACAACGCCGAGAACAC	1,711	60
*SOX4*-cds -R	CTCGAGAATAAAAGGTCGCCCCTGTCTA		
ChIP-F	GCCTTTGGAGTATTGTTTCCTTC	141	60
ChIP-R	GGAGCCAGACTTCATTCCTTTTAT		

F, upstream primer; R, downstream primer.

*IGF1R*, type 1 insulin-like growth factor receptor; QPCR, quantitative real-time polymerase chain reaction; *SOX4*, SRY-box 4; ChIP, chromatin immunoprecipitation.

**Table 2:** Synthetic oligo sequences

Name	Sequence (5′-3′)
miR-140 mimic	CAGUGGUUUUACCCUAUGGUAGU
	CUACCAUAGGGUAAAACCACUGU
miR-control	UUCUCCGAACGUGUCACGUTT
	ACGUGACACGUUCGGAGAATT
miR-140 inhibitor	CUACCAUAGGGUAAAACCACU
Inhibitor control	CAGUACUUUUGUGUAGUACAA
*IGF1R* siRNA	GGAAGCUCUUCUACAACUACGTT
	UAGUUGUAGAAGAGCUUCCAGTT
*SOX4* siRNA	GCUGGAAGCUGCUCAAAGACATT
	UCUUUGAGCAGCUUCCAGCGUTT
siRNA NC	AAGACAUUGUGUGUCCGCCTT

*IGF1R*, type 1 insulin-like growth factor receptor; *SOX4*, SRY-box 4.
